# Observation of Point-Light-Walker Locomotion Induces Motor Resonance When Explicitly Represented; An EEG Source Analysis Study

**DOI:** 10.3389/fpsyg.2018.00303

**Published:** 2018-03-12

**Authors:** Alberto Inuggi, Claudio Campus, Roberta Vastano, Ghislain Saunier, Alejo Keuroghlanian, Thierry Pozzo

**Affiliations:** ^1^Unit of Robotics, Brain and Cognitive, Istituto Italiano di Tecnologia, Center for Human Technologies, Genova, Italy; ^2^Sciences, Istituto Italiano di Tecnologia, Center for Human Technologies, Genova, Italy; ^3^Unit for Visually Impaired People, Istituto Italiano di Tecnologia, Center for Human Technologies, Genova, Italy; ^4^Laboratório de Cognição Motora, Departamento de Anatomia, Universidade Federal do Pará, Belém, Brazil; ^5^Institut National de la Santé et de la Recherche Médical, Cognition-Action-Plasticité Sensorimotrice, Université Bourgogne Franche-Comté, Dijon, France; ^6^Centro di Neurofisiologia Traslazionale, Istituto Italiano di Tecnologia, Ferrara, Italy

**Keywords:** event-related potentials (ERPs), biological motion perception, electroencephalography (EEG), motor resonance, embodied cognition, goal-directed action

## Abstract

Understanding human motion, to infer the goal of others' actions, is thought to involve the observer's motor repertoire. One prominent class of actions, the human locomotion, has been object of several studies, all focused on manipulating the shape of degraded human figures like point-light walker (PLW) stimuli, represented as walking on the spot. Nevertheless, since the main goal of the locomotor function is to displace the whole body from one position to the other, these stimuli might not fully represent a goal-directed action and thus might not be able to induce the same motor resonance mechanism expected when observing a natural locomotion. To explore this hypothesis, we recorded the event-related potentials (ERP) of canonical/scrambled and translating/centered PLWs decoding. We individuated a novel ERP component (N2c) over central electrodes, around 435 ms after stimulus onset, for translating compared to centered PLW, only when the canonical shape was preserved. Consistently with our hypothesis, sources analysis associated this component to the activation of trunk and lower legs primary sensory-motor and supplementary motor areas. These results confirm the role of own motor repertoire in processing human action and suggest that ERP can detect the associated motor resonance only when the human figure is explicitly involved in performing a meaningful action.

## Introduction

One of the most impressive properties of the human visual system is the possibility to perceive a moving subject starting from the observation of a poor visual input. The most famous class of such stimuli is the point-light display (PLD), which consists of moving dots recorded while attached to the limbs of a human walking actor (Johansson, 1973). PLD stimuli have been largely investigated, showing that it is possible to infer a human moving body also when the canonical form is inverted, surrounded by noise or even scrambled, by randomly modifying dot positions while preserving their kinematic (Blake and Shiffrar, 2007). Presently, manipulations on PLD shape represent the most investigated experimental factor, revealing that several cortical areas are specifically involved in decoding the unique spatial features of human body shape (Bonda et al., 1996; Downing et al., 2001; Peelen et al., 2006; Saygin, 2007; Jastorff and Orban, 2009; Matheson and McMullen, 2010; Grosbras et al., 2012).

PLD locomotion have been investigated under a wide range of experimental manipulations, but all characterized by displaying the point-light walker (PLW) as walking on a treadmill, that is, without any forward movement. This kind of stimuli, the locomotor action, display intransitive body movements composed of successive and infinite cyclical legs and arms motion. Considering that a prominent idea regarding biological motion recognition is related to the so-called “motor way of seeing” (Calvo-Merino et al., 2005; Orgs et al., 2015), being the visual perception of kinematic features tuned by motor representations (Viviani and Stucchi, 1992; Pozzo et al., 2006; Saunier et al., 2008), the presence of a without goal-oriented body displacements should evoke a different motor response in the observer brain. According to this, the visual input of an observed action would be mapped on to the observer's own motor repertoire through a direct-matching mechanism (Gallese et al., 1996; Rizzolatti et al., 2001), also called motor resonance. In the case of observing an artificial walker on the spot, the visual input should be more difficult to match with the motor repertoire and one may predict different cortical activities for natural and treadmill locomotion. More precisely, considering that action observation shares common neurophysiological basis with motor execution (Jeannerod, 2001; Mulder, 2007), plausible sources for the motor resonance process could be hypothesized from previous studies on locomotion observation (Malouin et al., 2003; Iseki et al., 2008; Orgs et al., 2015). The latter revealed the activation of legs primary motor areas and supplementary motor and dorsolateral premotor area. Since body translation in space is the natural and expected outcome of the locomotion action, we expect a stronger pattern matching between such action and the PLW stimulus, when its legs and trunk movements also induce its spatial displacement. Consequently, considering that centered walker proved to evoke motor related activity, at least when time-frequency analysis was employed (Pavlidou et al., 2014; Lange et al., 2015), translating canonical walker would induce a higher motor resonance compared to a centered one.

To explore this hypothesis, we designed a two-by-two task protocol showing both a canonical or scrambled walker that in half of the trials stayed with its barycenter in the middle of the screen and in the other half walked from the center to the screen periphery, and we recorded the event related potentials (ERP) in a population of healthy subjects. We opted for an ERP approach to compare our results to all the previous EEG papers on PLW observation. Following the existing literature on centered PLW decoding, we investigated the two main ERP components: the early N1 and the later N2. The former is implied in the low-level decoding of basic and fine-grained form and motion features, is expressed in bilateral occipito-temporal scalp region and is presumably generated in extra-striate ventral stream areas. The latter represents a second stage of a more advanced analysis, likely either related to the integration of form and action (White et al., 2014) or the recognition of potential social implication. This component is expressed in bilateral occipito-temporal scalp regions and is thought to be generated in the posterior part of the superior temporal sulcus (pSTS) (Jokisch et al., 2005; Krakowski et al., 2011). Since ERP components to translating PLW were never tested, we took into account the possibility to investigate further components according to the observed scalp maps. In such cases, we also performed a distributed source analysis in order to locate the cortical sources underlying such novel components.

## Materials and methods

### Subjects

Thirteen right-handed volunteers (seven females, mean age: 27, standard deviation: 3.5), with normal or corrected to normal vision, took part in this study. The present study has been conducted according to the principles expressed in the revised Declaration of Helsinki (World Medical Association General Assembly, 2008) and was approved by the local ethical committee of ASL-3 (“Azienda Sanitaria Locale,” local health unit) of Genova, Italy. All participants provided written informed consent before the experiment began.

### Experimental protocol: stimuli and task

**Figure 1:**
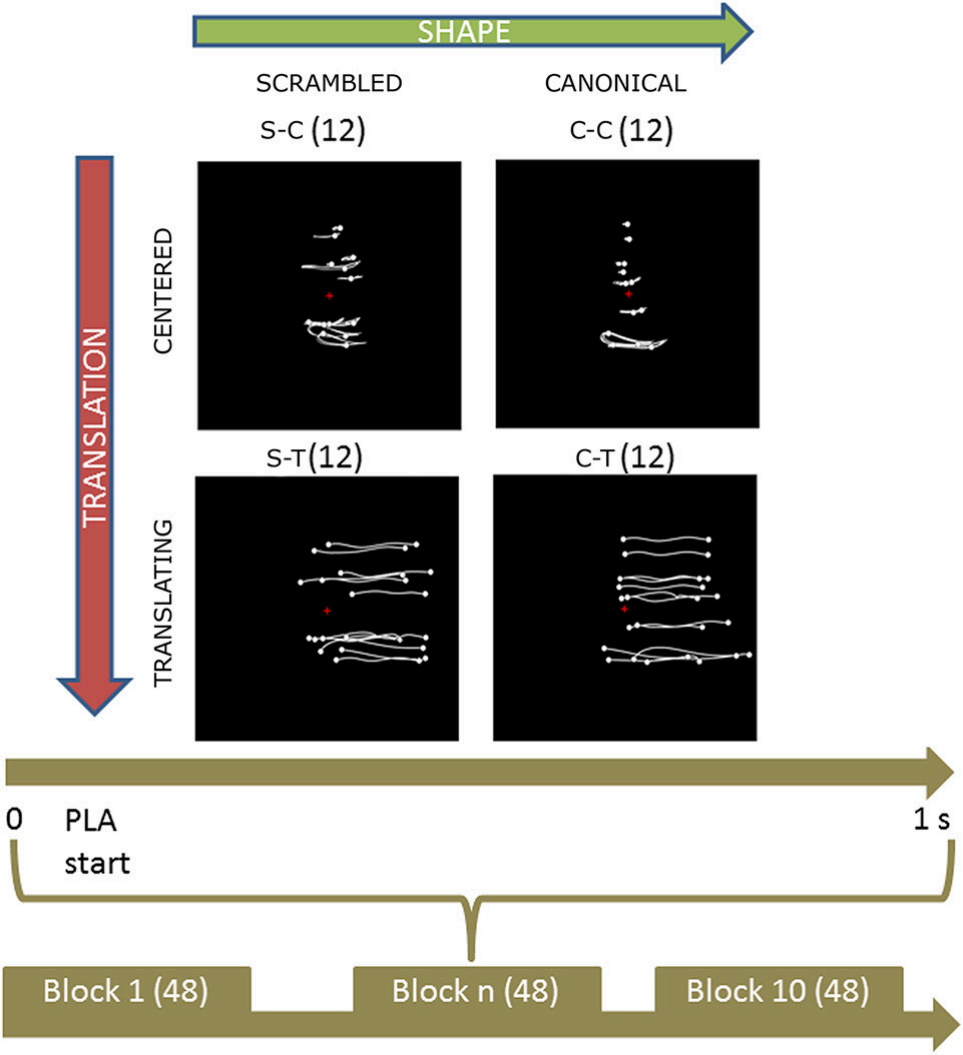
Experimental protocol and stimuli displays. Upper part shows the four categories of stimuli. Body joint trajectories (in white) are depicted on black screen for illustrative purposes but were not visible during the actual experiment. From left to right (upper horizontal green arrow), stimuli shape change from a scrambled structure to a real walker body structure. From top to bottom (left lateral yellow arrow), stimuli keep the same shape but change from a centered to a translated display. Abbreviations: S-C and C-C for scrambled and canonical centered PLW respectively; S-T and C-T for scrambled and canonical translating PLW. The trajectories of the markers during the whole point light animation (PLA) are shown. The lower part sums up the experimental protocol. During each of the 10 experimental blocks of 48 trials, every condition was randomly presented 12 times for 1 s. During each trial, subjects were told to anchor their gaze on the red cross displayed in the middle of the screen.

Participants were presented with PLW animations obtained by recording an actor walking naturally with a VICON Motion Capture System (10 cameras recording at 100 Hz). The actor had 13 passive infrared reflective markers placed at the main joints and other landmarks. Post-processed stimuli data were displayed using the Psychophysics Toolbox (Brainard, 1997) on an LCD monitor, with a refresh rate of 60 Hz. Point-lights were white against a black background. Four types of PLW stimuli were created: a centered canonical (CC), a centered scrambled (CS), a translating canonical (TC), and a translating scrambled (TS). The *CC* animation was built by translating all the dots of the *TC* animation by the opposite of the vector defining their center of mass with respect to the center of the screen. The *CS* animation was built by changing randomly the initial positions of the dots in the *CC* animation but keeping their velocity vectors unchanged; the dots' trajectories were constrained to remain inside the vertically oriented rectangle in which the *CC* animation was inscribed. The *TS* animation was built by changing randomly the initial positions of the *TC* animation in an analogous way. Translating stimuli, moved rightward from the monitor center. Each animation was 1 s long, allowing displaying two steps (that is one full locomotor cycle). All translating animation moved rightward. Experimental protocol is summarized in Figure 1. During the experiment, participants were sitting comfortably in a darkened room in front of the monitor where PLWs were displayed, ~60 cm apart. Consequently, centered and translating conditions were observed with a visual horizontal angle between 0 and 5.7° (also considering the upper limbs dots' oscillations) and 0–18.9° respectively; stimulus vertical size was 9.5 degree. The experiment was organized in 10 blocks composed by 48 PLW animations (12 of each of the four types, for a total of 120 trial for each experimental condition) presented in pseudo-random order, with an inter-trial interval (ITI) varying randomly between 2 and 4 s. Participants were asked to maintain their gaze fixed toward the screen center during PLW observation, to help them a fixation cross was displayed in the screen center during the ITI period.

We are aware that the chosen design may induce smooth pursuit eye movements toward translating stimuli, thus possibly biasing our comparisons. Nevertheless, in our opinion, alternative scenario, matching eye movements, would have introduced biases even more difficult to compensate for. For instance, a straightforward, in principle, way to represent translation along with matching eye movements, the one showing a translating background, would have represented an ecological stimulus only assuming that also the observer had moved in the same direction. Consequently, the resulting motor related activity could have been a correlate of the sensorimotor simulation of the *observer*'s implicit translation, rather than of the *observed* explicit translation. We thus opted for the most ecological locomotion representation and paid special attention to compensate for the effect of eye movements on ERP results.

### Attention task

In each block, a random (between 2 and 4) number of animations (odd trials) changed color from white to green for 250 ms. After a random (between 1 and 3) number of trials, participants were asked to indicate which condition had previously changed its color, by pressing a key number between 1, 2, 3, and 4 (indicating respectively CC, CS, TC, and TS stimuli). Each participant learned the correspondence between these keys and the stimuli during a training session before starting the actual experiment. Subjects did not receive any feedback on their performance during the experiment. Odd trials were later discarded from analysis.

### Data recording and preprocessing

The electroencephalogram (EEG) was recorded from 62 Ag/AgCl active electrodes (actiCAP, Brain Products, Munchen, Germany) placed on the scalp, mounted on a cap according to the international 10–20 system. Reference and ground electrodes were set at the place of FCz and AFz electrodes respectively. The EEG was amplified with two BrainAmp MR plus amplifiers (Brain Products), digitized at 1,000 Hz. Impedances of all electrodes were kept below 10 kOhms. Raw EEG signals were band-pass filtered between 0.15 and 45 Hz through a Butterworth filter as implemented in Brain Vision Analyzer software (Brain Products GmBH, Gilching, Germany). An Ocular ICA correction was applied to remove eye-blink related artifacts. Data were down-sampled to 250 Hz, then imported into EEGLAB software and referenced to a common average. No bad electrodes were found, no trials had to be discarded. Epochs, from −400 to 1,000 ms with respect to stimulus presentation, were baseline-corrected (from −400 to 0) and then averaged in order to produce the ERP of each four experimental condition.

### Components assessment

We calculated the peaks amplitudes of the two most prominent (Krakowski et al., 2011; White et al., 2014) ERP components (N1 and N2) according to a well-established method (Hirai et al., 2003; Jokisch et al., 2005; Krakowski et al., 2011; White et al., 2014). In the spatial domain, we created a cluster by averaging the amplitudes of those occipito-temporal electrodes commonly used in the literature to model such component. In the temporal domain, (i) we individuated, at the group level, a preliminary time window where each component did express itself and (ii) calculated the individual peak, then (iii) we averaged the cluster amplitudes within a 20 ms-wide time window centered on that peak. We finally obtained one measure for each subject/condition/component that was then statistically investigated. Since translation was expected to modify brain response and no previous ERP studies were available for reference, we looked for further components by visually exploring the scalp maps and by performing a cluster-based permutation analysis where the significance probability was calculated by means of the so-called Monte Carlo method (Maris and Oostenveld, 2007). The latter analysis results were used to identify the new components' cluster electrodes and group preliminary time window. Novel components were then analyzed with the same methods used for N1 and N2 ones.

### Eye movement analysis

Although subjects were asked to keep their gaze on the screen center, since half of the stimuli translated along the screen, smooth pursuit eye movements were expected to may occur during translating stimuli observation. As a possible consequence, part of the measured cortical activity might have been related to piloting such movements rather than to processing the stimulus content. In order to may correct our data for this effect, we calculated the magnitude of eye movements as the time course of the absolute value of the electric potential at electrode AF7 referenced to AF8 [hEye = abs(AF7–AF8)] and we then calculated its average value in correspondence to each ERP component's peak (hEye_N1_, hEye_N2_, hEye_N2c_), using the same previously defined time windows. A linear fit was then estimated between each hEye value and ERP component amplitude and, when it resulted significant, the estimated slope and intercept values were regressed out from the latter data. AF7 and AF8 have been previously considered among the most significant forehead electrodes to detect eye movements (Belkacem et al., 2013) and used to detect eye activity during a covert horizontal tracking task (Makin et al., 2012). According to their position and to the pointing direction of the corneo-retinal dipoles in the eyes (Croft and Barry, 2000), their amplitudes were expected to deflect coherently with the direction of an horizontal eye movement and thus correlate with the time course of an ordinary horizontal electro-oculogram.

### Statistical analysis

For each ERP component, a factorial 2 × 2 model, investigating the effect of the within subjects TRANSLATION (centered, translating) and SHAPE (canonical, scrambled) factors and their interactions, was created in EEGLAB and analyzed through a permutation analysis (Delorme, 2006) employing 100000 permutations. *Post hoc* analyses were performed through paired *t*-test, corrected for multiple comparisons through the false discovery rate (FDR) approach within each component. The same factorial model was tested on the three hEye channel values, calculated in correspondence to the three components (hEye_N1_, hEye_N2_, hEye_N2c_). The classic *p* < 0.05 threshold was used.

### Source analysis

In order to reconstruct the cortical generators of those ERP components affected by the translation factor, we employed a distributed sources analysis using the Brainstorm software (Tadel et al., 2011). Cortical current source distribution within the brain was represented through 15,002 elementary dipoles obtained by sampling a tessellated cortical mesh template surface derived from the standard 1 mm resolution brain (Colin27) of the Montreal Neurological Institute. Since the individual MRIs were not available, and thus the dipole orientations derived from the template could not in any way approximate the actual brain geometry, dipole orientations were not fixed normal to the cortex surface but were let free to assume whichever orientation. The EEG forward modeling of volume currents was completed with a three-layers (head, outer, and inner skull) symmetric boundary element model (BEM) generated with OpenMEEG (Gramfort et al., 2011). A diagonal noise covariance matrix was computed for each participant using the pre-stim interval to estimate the sensors variance. Sources intensities were estimated through a depth-weighted minimum norm approach (Baillet et al., 2001). This technique has been shown to be robust to noise in recorded data and head model approximations with fair spatial resolution (Baillet et al., 2001), and the depth weighting used in this approach alleviates the natural bias of basic minimum norm estimation approaches toward superficial currents. Brainstorm's default parameter settings have been used for both source reconstruction and BEM creation. Sources data were then post-processed as following: (i) since we were interested in evaluating source intensity differences, the norm of the vectorial sum of the three orientations at each vertex was calculated for each time-point; (ii) source data were reduced in the time domain by applying the same procedure used for ERP data. Each source activity was averaged across five time-points (20 ms), centered on the peak of the ERP component to be reconstructed. Finally, pairwise comparisons between two experimental conditions were investigated with paired *t*-test and results were corrected for multiple comparisons according to two different approaches: a classical *p* = 0.05 FDR corrected threshold and a more liberal *p* = 0.0005 uncorrected one.

## Results

### Attention task

Participants were able to correctly discriminate the 91.8 % of the stimuli (CC: 92.3%, TC: 90.8%, CS: 92.1%, TS: 92%).

### Visual inspection of ERP data

**Figure 2:**
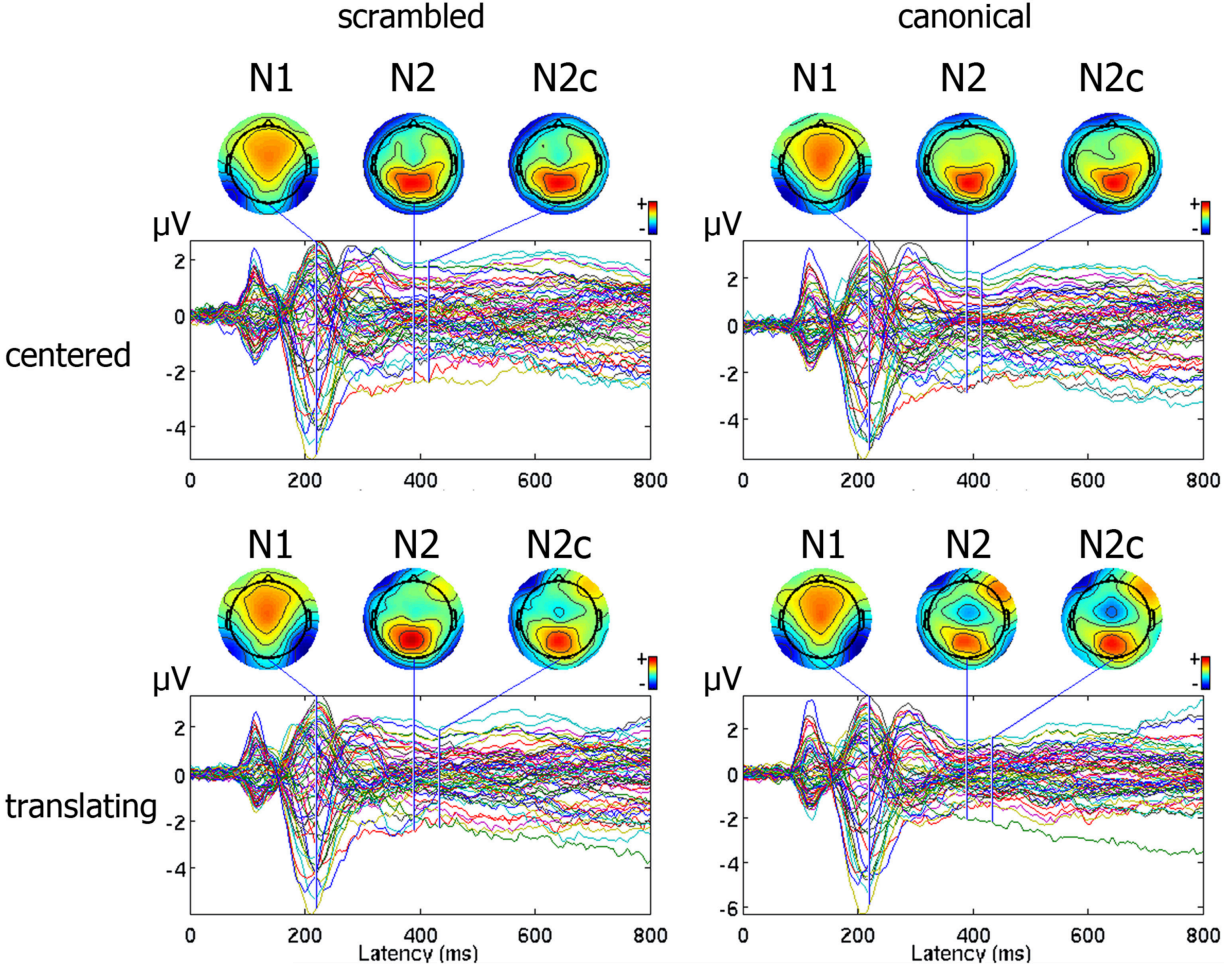
Representation of ERP pattern. Data are represented as a butterfly plot, showing scalp maps at the onset of the classical N1 and N2 components and of the novel central N2, here labeled as N2c, and observed around the vertex only in the translating centered condition.

**Figure 3:**
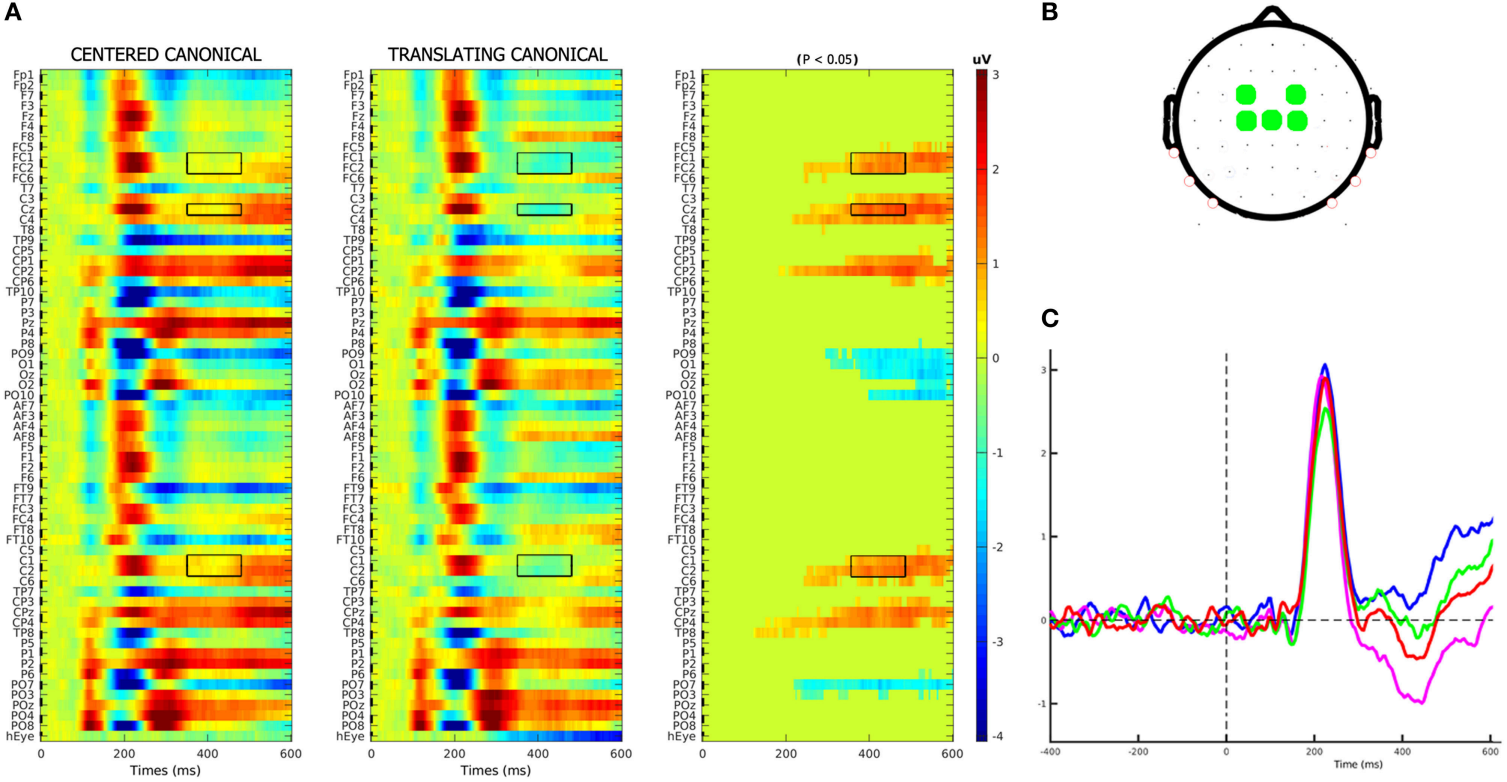
N2c component identification. **(A)** Results of cluster-based permutation analysis of the CC–TC conditions' contrast. In first two columns, the two conditions' group-averages are displayed. In the third column, we represent their amplitude differences (in μV), in those channels-latencies pairs where a significant (*p* < 0.05) difference was found. In accordance with the scalp topography observed for TC condition, the electrodes cluster used to model the negative N2c component was composed by those electrodes that had negative (blue) values in TC condition and positive (red) in CC ones. Selected electrodes-latencies were marked by a black rectangle. **(B)** Fronto-central electrodes, defined according to the above criteria, used to compose the N2c cluster. **(C)** Plot of the N2c cluster's group-average in each condition.

An average number of 4.5 ± 1.2 trials for each subject were discarded from the analysis for movement-related artifact. Visual exploration of the scalp maps revealed, together with the two classical N1 and N2 components, a fronto-central negativity in canonical translating PLW, which was completely missing in centered stimuli (Figure 2). We thus performed a cluster-based permutation analysis (Figure 3A) over our main pairwise comparison of interest, the translation effect within canonical PLW, which revealed a set of electrodes that resulted significantly different (*p* < 0.05). To estimate such novel component, we created a cluster composed by those significantly different electrodes that had positive values in CC and negative ones in TC (C1, C2, Cz, FC1, and FC2). We then plotted its temporal evolution (Figure 3C) and found a peak around 435 ms. For its negative polarity and its latency, <40 ms after the N2, we called this component central N2 (N2c). Table 1 summarizes the electrodes contained within each cluster and the temporal window used to search for the individual peak.

**Table 1:** Electrodes clusters composition.

**Component**	**Cluster name**	**Electrodes list**	**Time window**
N1	Ventral (inferior occipito-temporal)	PO8, P8, TP8, PO7, P7, TP7	180–250
N2			360–450
N2c	Fronto-Central	C1, C2, Cz, Fc1, FC2	380–480

*List and composition of the electrodes clusters used to model the N1 and N2 components in centered PLW. An additional cluster, defined according to cluster-based permutation analysis, was kept in consideration for modeling the N2c component present only in translating canonical (C-T) PLW. The time window column reports the interval where the component individual peak was looked for*.

### Eye channel data

**Figure 4:**
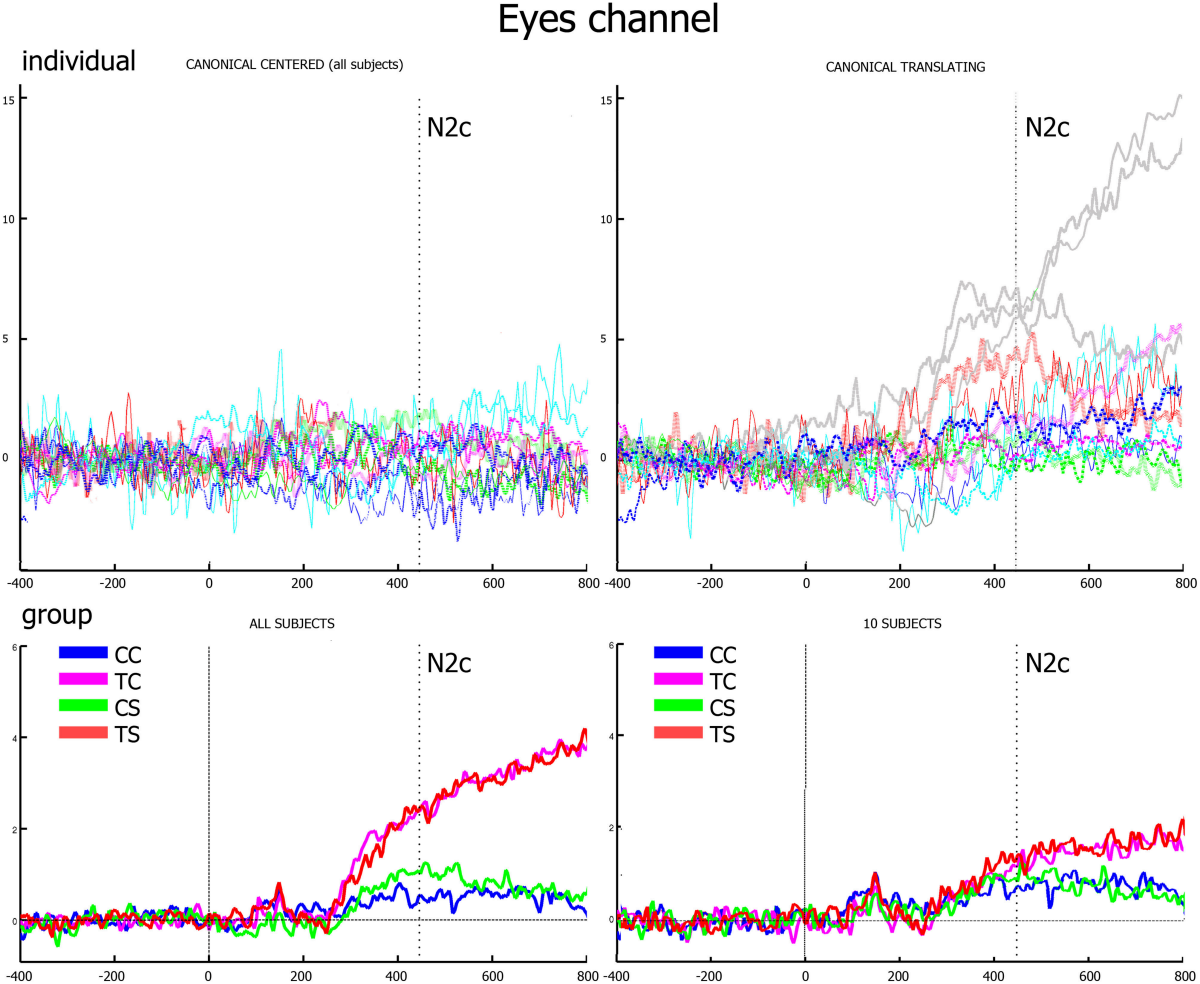
Eye channel traces. In the upper row, the individual traces of canonical center **(left)** and canonical translating **(right)** conditions clearly show that only three subjects smoothly pursued the translating stimuli (gray lines). In the lower row, group averages of the whole group **(left)** and of the not-pursuing subjects group **(right)** are displayed. The vertical dotted line represents the N2c latency when, in the latter group, eye movements to translating and centered conditions are almost equivalent.

**Figure 5:**
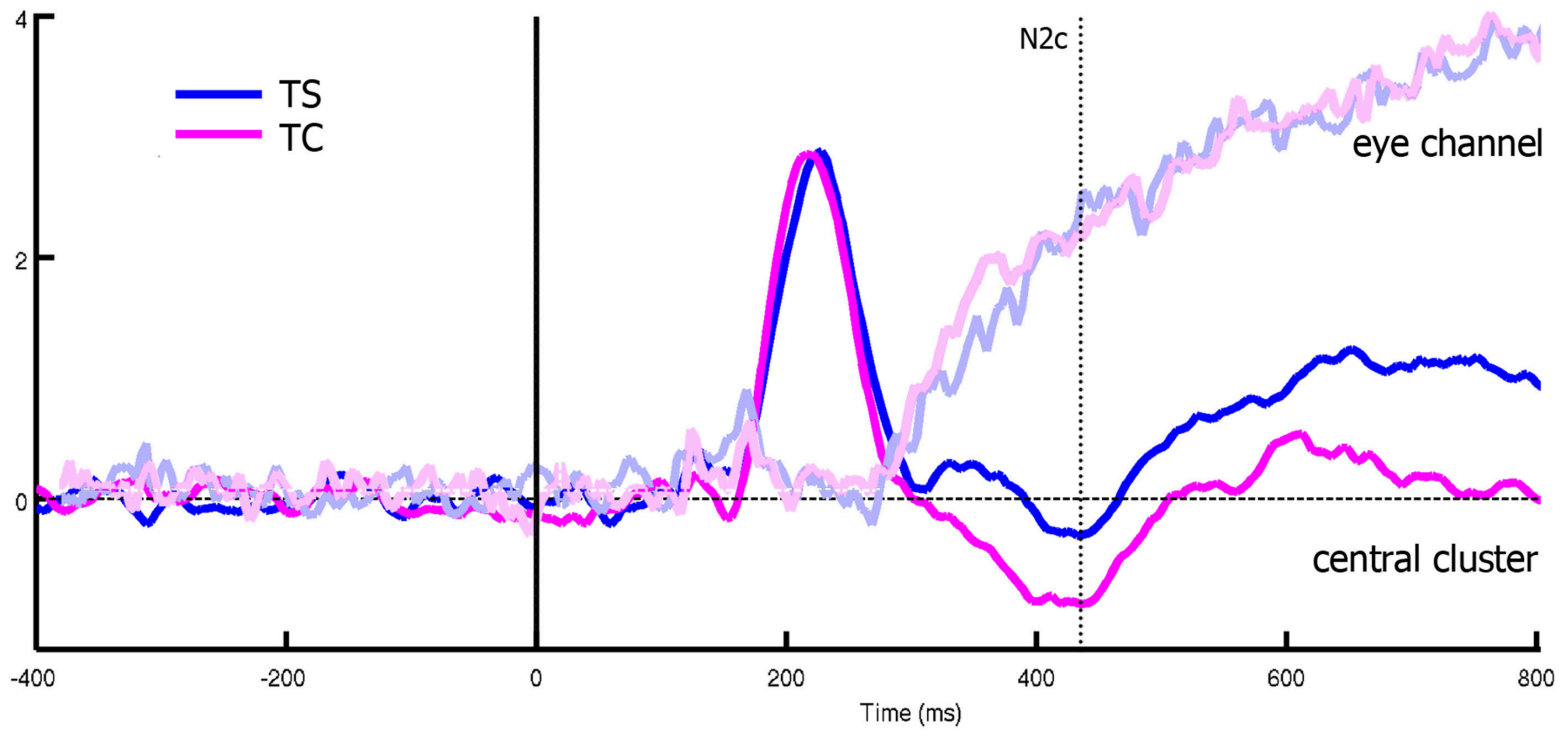
Differences between eye channel and central cluster within translating conditions, considering all the subjects. While the two eye-channel traces overlaps, central cluster one showed a significant deviation that cannot be thus related to eye muscles effects.

The analysis on rectified eye channel values (hEyes) showed that, from 250-300 ms, only three subjects smoothly pursued the translating stimuli (gray curves of the two top panels of Figure 4). At the group level, such progressively increasing separation between translating and centered conditions resulted significant only in the correspondence of the N2c [main effect of translation: *F*_(1, 12)_ = 5.48, *p* = 0.037, η_2*p*_ = 0.31, bottom-left panel of Figure 4]. The shape effect and the interaction between the two were not significant. Repeating instead the analysis on eye channel values for the remaining 10 subjects (successively referred as the not-pursuing group), the translation effect during the N2c component disappeared [*F*_(1, 12)_ = 1.34, *p* = 0.27, η_2*p*_ = 0.12; bottom-right panel of Figure 4]. No significant correlations, either considering the whole group or the not-pursuing one, were found among hEye_N1_, hEye_N2_, hEye_N2c_ values and the corresponding ERP components amplitudes; hence the latter data were not corrected for eye movement. Figure 5 clearly shows, for example, the absence of any relationship between hEye channel traces and central cluster amplitudes: while the former traces during the two translating conditions almost overlapped, the ERP traces in the central cluster were clearly different.

### ERP data

Statistical analyses were first performed on the whole group. Successively, they were repeated for the not-pursuing group.

#### Main effects of shape and translation

**Figure 6:**
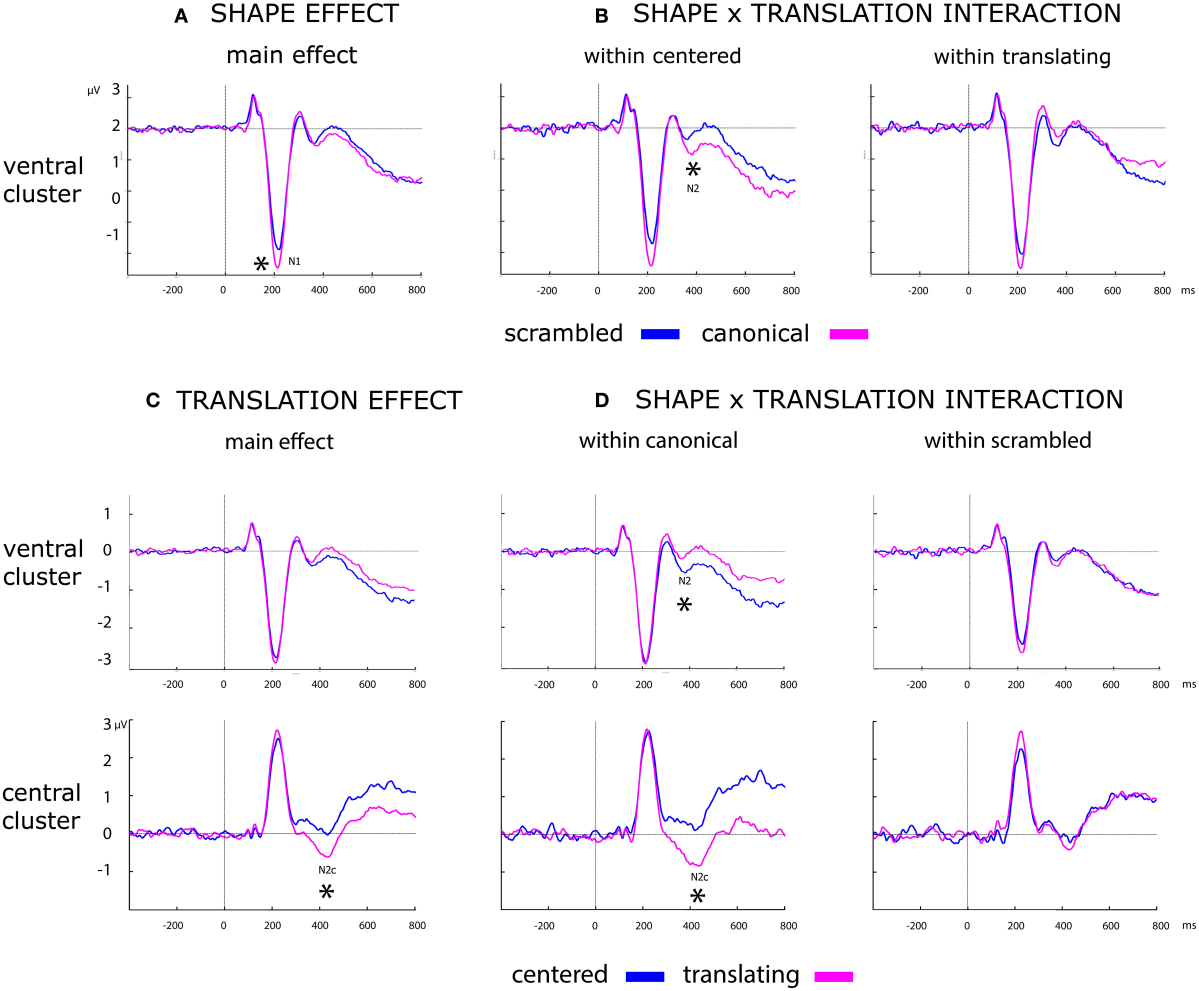
Effect of shape, translation and their interaction over ERP data. Black asterisks below the figures represent: **(A)** main effect of shape during N1. **(B)** *Post-hoc* of the shape × translation interaction, showing the within centered only effect of shape during N2. **(C)** Main effect of translation over N2c component. **(D)** *Post-hoc* of the shape × translation interaction, showing the effect of translation during N2 and N2x only within canonical PLW.

Results of the analysis of the main effects and their interactions are summarized in Table 2 and displayed in Figure 6.

**Table 2:** Main effects and interactions.

**Comp**.	**Shape effect**	**Translation effect**	**Interactions**
N1	*F*_(1, 12)_ = 4.54, *p* < 0.001; η_2*p*_ = 0.43		
N2			*F*_(1, 12)_ = 13.84, *p* = 0.003; η_2*p*_ = 0.34
N2c		*F*_(1, 12)_ = 7.50, *p* = 0.001; η_2*p*_ = 0.43	*F*_(1, 12)_ = 7.19, *p* = 0. 019; η_2*p*_ = 0.42

*Results of non-parametric bootstrap ANOVA: Main effects and interactions. The η_2p_ symbol represents the partial eta squared measure of the ANOVA effect size*.

##### Shape

A more negative deflection of N1 was found for canonical with respect to scrambled PLW. Results are summarized in the left column of Table 2 and displayed in Figure 6A.

##### Translation

A stronger negative deflection of N2c was found for translating with respect to centered PLW. Results are summarized in the right column of Table 2 and displayed in Figure 6C.

#### Interactions between shape and translation

##### Shape effect within centered and translating conditions

*Post-hoc* analysis results are summarized in Table 3. N2 confirmed its sensitivity to the shape factor only within centered PLWs, resulting more negative for the canonical compared to scrambled conditions. The N2c showed a more negative deflection in canonical compared to scrambled PLW, only when both translated.

**Table 3:** *Post-hocs* analysis of shape and translation factors.

**Comp**.	**Within centered (C-C vs. S-C)**	**Within translating (C-T vs. S-T)**
**SHAPE**		
N2	*t*_(12)_ = 3.66, *p* = 0.005	
N2c		*t*_(12)_ = 2.23, *p* = 0.005
**TRANSLATION**		
**Comp.**	**Within canonical (C-T vs. C-C)**	**Within scrambled (S-T vs. S-C)**
N2	*t*_(12)_ = −2.70, *p* = 0.019	
N2c	*t*_(12)_ = 5.21, *p* < 0.0005	

*Post-hocs were performed through paired t-test, FDR corrected within each component. Single conditions are named according to the following convention: shape-translation; C-C, canonical-centered; C-T, canonical-translating*.

##### Translation effect within canonical and scrambled conditions

*Post-hoc* analysis results are summarized in Table 3. Both ventral and central N2 resulted sensitive to translation factor, but only considering canonical PLW.

#### Analyses on the not-pursuing group

All the analyses were repeated for the not-pursuing group of 10 subjects. A same ERP negative deflection on scalp topography could be observed in absence of eye movements (Figure S1) and statistical analyses, summarized in supplementary Table 4, completely confirmed the whole group results. These supplementary analyses suggest that eye movements did not influence our findings.

**Table 4:** Statistical analysis on the not-pursuing group.

**MAIN EFFECTS AND INTERACTIONS**			
**Comp**.	**Shape effect**	**Translation effect**	**Interactions**
N1	*F*_(1, 12)_ = 14.23, *p* = 0.0011; η_2*p*_ = 0.51		
N2			*F*_(1, 12)_ = 11.12, *p* = 0.0027; η_2*p*_ = 0.28
N2c		*F*_(1, 12)_ = 11.9, *p* = 0.0004; η_2*p*_ = 0.34	*F*_(1, 12)_ = 13.25, *p* = 0. 0042; η_2*p*_ = 0.62
***POST-HOCS*** **ANALYSIS OF SHAPE AND TRANSLATION FACTORS**			
**Shape**	
**Comp**.	**Within centered (C-C vs. S-C)**	**Within translating (C-T vs. S-T)**	
N2	*t*_(12)_ = 2.67, *p* = 0.0011	
N2c		*t*_(12)_ = 3.01, *p* = 0.0007	
**Translation**	
**Comp**.	**Within canonical (C-T vs. C-C)**	**Within scrambled (S-T vs. S-C)**	
N2	*t*_(12)_ = −2.01, *p* = 0.0038	
N2c	*t*_(12)_ = 3.98, *p* = 0.0005	

*Upper panel: Results of non-parametric bootstrap ANOVA: Main effects and interactions. The η_2p_ symbol represents the partial eta squared measure of the ANOVA effect size. Lower panel: Post-hocs were performed through paired t-test, FDR corrected within each component. Single conditions are named according to the following convention, shape-translation; C-C, canonical-centered; C-T, canonical-translating*.

#### Source analysis

**Figure 7:**
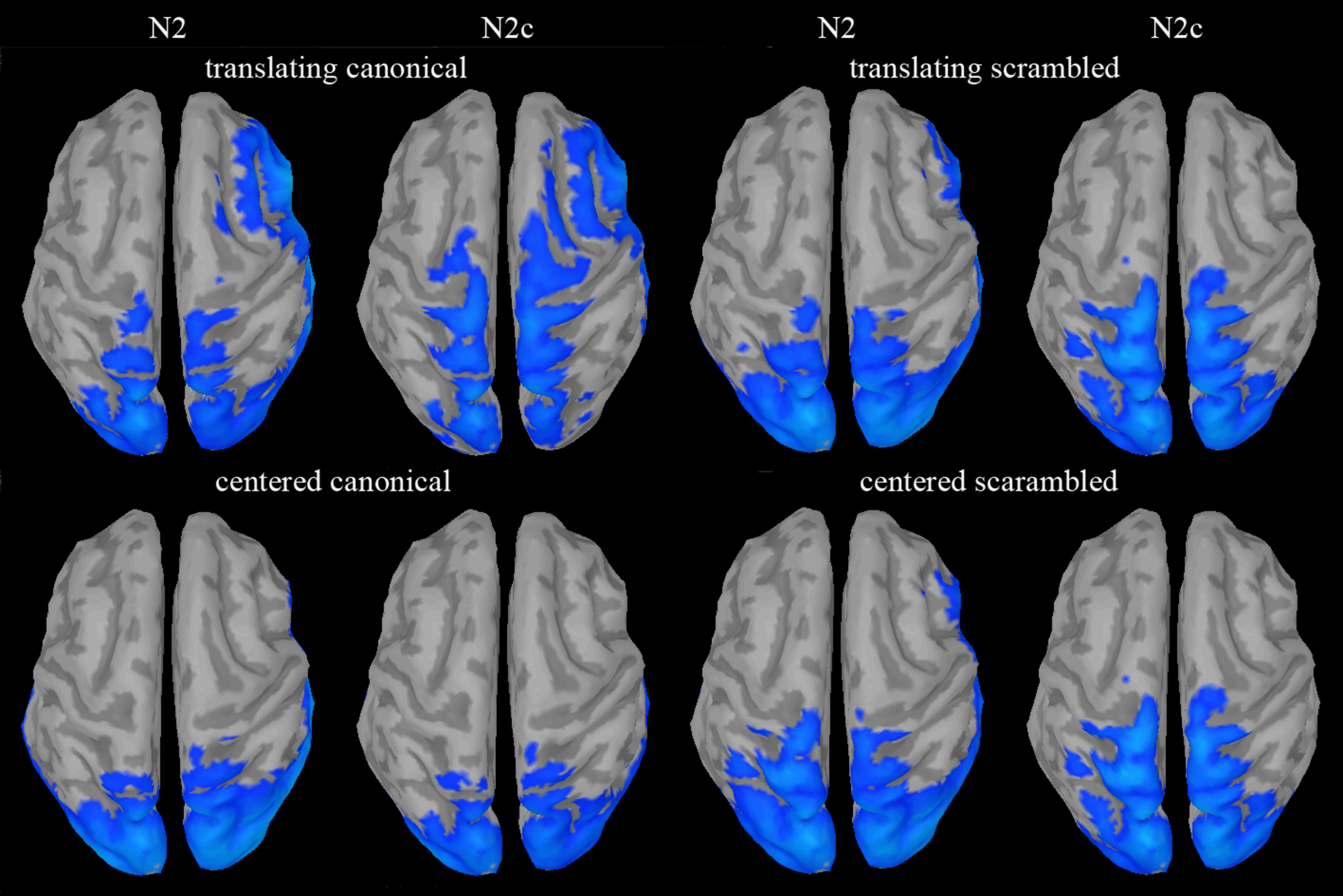
Source analysis of N2 and N2c components. A clear sensorimotor and supplementary motor activity, with respect to the baseline period, could be found only during N2c in the translating canonical condition.

**Figure 8:**
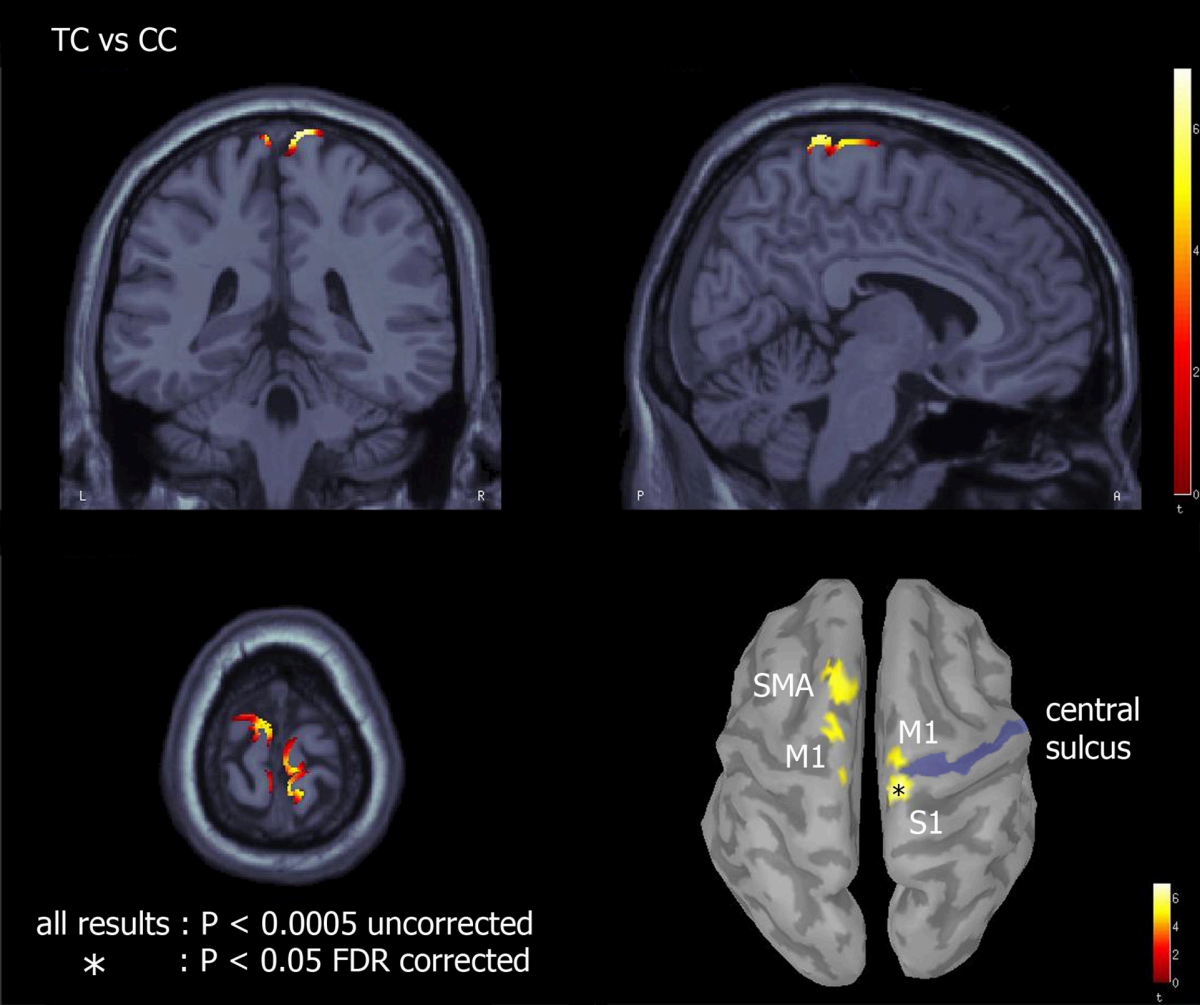
Effect of translation within canonical walkers. Source analysis of the paired *t*-test between TC and CC showed areas of increased activation for translating with respect to centered condition. The asterisk represents brain regions resulting significant with *p* < 0.05 and FDR corrected for multiple comparisons; the remaining regions with *p* < 0.0005 uncorrected.

Source analysis was only used to reconstruct the cortical generators of the novel N2c component and to assess if such component could be considered a novel one with respect to N2. An explorative source analysis of the brain patterns underlying the N2 and N2c components (Figure 7) revealed a different set of generators for the two components when the translating PLW also preserved the human figure. To statistically measure the effect of the translation factor within both canonical and scrambled PLW, for each subject and condition, we used the individual ERP peak latencies of the central cluster to determine the center of the 20 ms wide window used to average the sources activity. The activity of the individually defined single time-point was compared between conditions. According to the multiple comparison correction employed, we found an increased activation to translating PLW only within canonical stimuli. Using the FDR correction, we found a focused activation spot overlaying lower limbs primary somatosensory cortex [MNI coordinates: x = 7, y = 39, z = 84, *t* = 6.99), while using a more liberal threshold (*p* < 0.0005 uncorrected) we found a larger active region embracing also primary and supplementary motor areas almost bilaterally. Figure 8 shows the resulting pattern according to the two employed corrections. No sources resulted sensitive to translation in the scrambled conditions.

## Discussion

In the present paper, we wanted to test the role of translation on visual motion processing of locomotion, by displaying a PLW moving or not across the screen combined with scrambled or human body structure. Previous studies depicted PLW on a treadmill, staying fixed in the center of the screen. According to recent theories on action perception (Gallese et al., 1996; Rizzolatti et al., 2001; Calvo-Merino et al., 2005; Orgs et al., 2015), we speculated that the observation of a complete representation of the locomotion, like that depicting an actual displacement of the human agent, could evoke a different brain pattern compared to the perception of an artificial centered PLW.

### Shape and kinematic integration

The first result of our study is the N1 and N2 selectivity to human body structure. This finding replicates previous EEG and MEG studies reporting a stronger negativity for canonical with respect to scrambled walker around both the occipito-temporal N1 and N2 components (Hirai et al., 2003, 2005; Jokisch et al., 2005; Krakowski et al., 2011; White et al., 2014) and confirms that these components are associated to the recognition of human body shape.

The main result of our study was the presence of a novel negative deflection during translating canonical PLW observation. Since it clearly emerged over central electrodes (Figure 2) and peaked only <40 ms after the classical ventral N2, we labeled it N2c. The *post hoc* analysis of the significant interaction between translation and shape showed that the amplitude of the N2c component was significantly larger during canonical translating condition compared to either canonical centered and scrambled translating ones. Thus, the simultaneous display of both human figure's local motion and spatial displacement seemed to boost brain regions' activity where locomotion-related cortical motor centers reside.

We speculate that N2c would represent an integrative encoding of the two motor components usually present during natural daily life locomotion perception: dots' structured local oscillatory kinematics (following the two-third power law) and displacement kinematic. If this hypothesis was verified, it may explain the lack of N2c in previous ERP investigations for which protocols displayed only body limbs oscillations without any horizontal PLW displacement. Further, a visual separation between shape and kinematic is only possible using artificial experimental protocols and rarely visible during normal daily life where limbs oscillation and translation are always associated. The very few probability to see a walker gliding on the ground probably reflects the strong visual effect produced by a “moonwalk” dance. With respect to phylogenesis, locomotion on the spot, as an aberrant motion for an alert predator, probably occupied a different status in the motor repertoire requiring specific neural circuitry besides the motor resonance network.

In fact, an evident behavioral difference between natural and on the spot locomotion is that in the latter condition successive footsteps are passively initiated as a reaction to the backward displacement of the support surface, which limits the voluntary component of the motion initiation and walker alertness.

At last, the functional dependency of kinematic from body structure becomes clearer when considering biomechanical variables of locomotion. Indeed, mechanical transformation of ground reaction force and gravity into body forward acceleration is only possible through a particular body geometry, that is the so called “inverted pendulum” biomechanical configuration (Winter, 2009). Thus, scramble displays provide body shapes incompatible with kinetics laws producing walking movement.

Source analysis on the translation effect within both canonical and scrambled walkers revealed that to a more negative N2c over the scalp vertex corresponded an increased activation of the mesial sensory cortex (using the more conservative threshold). Using a more liberal statistic threshold we found the same effect in regions belonging to the mesial primary and supplementary motor areas, only in the within canonical conditions (Figure 8). Although locomotion neural control mostly occurs at either spinal [39], subcortical [40] and cerebellar [41] levels, the present cortical activation is well known (Lajoie and Drew, 2007; Fling et al., 2014) [18]. Central primary motor (legs and trunks), premotor and supplementary motor areas are in fact involved in modulating the activity of the former neural structures to implement locomotion. Supplementary motor area is implicated in locomotion initiation [18] while posterior parietal cortex in dealing with obstacles management [19].

Motor activity was found few tens of milliseconds after the N2 component which is thought to be generated by pSTS (Jokisch et al., 2005; Krakowski et al., 2011). The latter area, besides being implicated in shape decoding, is part of the action observation network (AON) and contributes with the mirror neuron system (Kilner, 2011; Avenanti et al., 2013) in extracting the observed action meaning (Jokisch et al., 2005). Since N2 and N2c components showed similar *post-hoc* results and their peak activities are separated by only few tens of milliseconds, we thus support the possibility that they reflect the cooperation between pSTS and locomotion-related motor areas in encoding goal-oriented locomotion pattern. A similar cooperation, has been already hypothesized for biological visual flow processing (Saunier et al., 2013). Source analysis on the N2 component was actually tested, but it did not produce any significant or nearly significant activation, neither comparing TC vs. CC conditions nor comparing each condition vs. their baseline period (Figure 7). Interestingly, Ulloa et al. (Ulloa and Pineda, 2007) found similar sensorimotor activity when observing human actions with limited forward displacement but all goal oriented (like kicking or doing the jumping jack movement). Altogether, this suggests that, in addition to the translation of the visual scene, the transitive dimension of the observed motion would facilitate the motor resonance mechanism.

The present motor brain response, recorded during the observation of natural translated locomotion compared to walking on the spot, has in our opinion interesting implications, as it might explain previous behavioral and psychophysics results. The idea that a natural locomotor pattern would better resonate with the observer's motor repertoire agrees with a recent finding showing better visual discrimination of a translating walker compared to a translating non biological figure, while in the absence of translation (a centered walker vs. a rotating geometrical figure) such difference could not be observed (Hiris, 2007). Similarly, subjects that learned to execute new movements, through verbal instructions, demonstrated later a better skill in visually discriminating those same movements (Casile and Giese, 2006). Also in support to this, when the link between action and perception system is interrupted due to lower limbs deafferentation (e.g., paraplegic patients), somatotopic sensorimotor reorganization and neural atrophy, observers show a poor performance in detecting and discriminating the direction of a translating PLW (Arrighi et al., 2011). At last, even if visual preference and stimulus discrimination cannot be considered a-priori related, the importance of an existing locomotor repertoire in processing a translating human figure is supported by newborns behavior. Two-days newborns in fact, result equally attracted by both a human figure and a cloud of random moving dots when both translated (Bidet-Ildei et al., 2014). In conclusion, a preferential visual processing for the human walk seems to evolve according to the human motor repertoire.

### Potential role of eyes movements

The locomotion action was rendered through the simplest and most ecological way by displacing horizontally the PLW across the screen. Although participants were asked to fix the screen center, three of them did pursue the translating stimuli. Moreover, assuming that in the absence of a fixation point, the expected behavior is to follow the translating stimulus, participants of the not-pursuing group possibly corrected this by saccades. Both smooth pursuit and saccade activity are known to produce strong potential deflections on neighbors' electrodes as an inverse function of their distance. This kind of activity superimposes itself on real brain activity and may have determined a bias in our analysis, creating differences between centered and translating conditions and thus invalidating our comparisons. Additionally, it exists the possibility that translating targets may unselectively recruit more attentional resources compared to the centered one. Nevertheless, several facts suggest that our N2c activity was not biased by these phenomena. First, significant ERP differences exist between the two translating conditions, while their eye-channel traces almost coincided (Figure 5). Such independence between eye movements and the brain pattern associated to the N2c component was further confirmed by the absence of any significant linear fit between them. Second, the effect of the smooth pursuit eye movement or the alternative saccade correction can be discarded considering that the analysis on not-pursuing group coincided with the whole group's one.

## Conclusion

Walking on a treadmill creates a conflicting sensorimotor context where voluntary body movement (legs and arms oscillations) do not induce self-motion perception. Consequently, when displayed, it triggers different visual perception processes, a result that should be kept into consideration when designing ecological stimuli for investigating brain processes related to biological motion perception. At last, once such result had been confirmed in a larger population of healthy subjects and patients, the N2c peak, presently uncovered, could represent a pertinent clinical marker for evaluating the effect of locomotion-related rehabilitation procedures.

## Author contributions

AI: analyzed and interpreted the data, drafted the manuscript; CC: analyzed the data; RV: analyzed and drafted the manuscript; GS: interpreted the data and drafted the manuscript; AK: recruited the subjects, recorded and analyzed the data; TP: designed the task, interpreted and drafted the manuscript.

### Conflict of interest statement

The authors declare that the research was conducted in the absence of any commercial or financial relationships that could be construed as a potential conflict of interest.

## References

[B1] Arrighi R. Cartocci G. Burr D. (2011). Reduced perceptual sensitivity for biological motion in paraplegia patients. Curr. Biol. 21, R910–R911. 10.1016/j.cub.2011.09.048 22115454

[B2] Avenanti A. Candidi M. Urgesi C. (2013). Vicarious motor activation during action perception: beyond correlational evidence. Front. Hum. Neurosci. 7:185. 10.3389/fnhum.2013.00185 23675338 PMC3653126

[B3] Baillet S. Riera J. J. Marin G. Mangin J. F. Aubert J. Garnero L. (2001). Evaluation of inverse methods and head models for EEG source localization using a human skull phantom. Phys. Med. Biol. 46, 77–96. 10.1088/0031-9155/46/1/306 11197680

[B4] Belkacem A. N. Hirose H. Yoshimura N. Shin D. Koike Y. (2013). Classification of Four Eye Directions from EEG Signals for Eye-Movement-Based Communication Systems. J. Med. Biol. Eng. 34, 581–588. 10.5405/jmbe.1596

[B5] Bidet-Ildei C. Kitromilides E. Orliaguet J.-P. Pavlova M. Gentaz E. (2014). Preference for point-light human biological motion in newborns: contribution of translational displacement. Dev. Psychol. 50, 113–120. 10.1037/a0032956 23668800

[B6] Blake R. Shiffrar M. (2007). Perception of human motion. Annu. Rev. Psychol. 58, 47–73. 10.1146/annurev.psych.57.102904.190152 16903802

[B7] Bonda E. Petrides M. Ostry D. Evans A. (1996). Specific involvement of human parietal systems and the amygdala in the perception of biological motion. J. Neurosci. 16, 3737–3744. 8642416 10.1523/JNEUROSCI.16-11-03737.1996 PMC6578830

[B8] Brainard D. H. (1997). The psychophysics toolbox. Spat. Vis. 10, 433–436. 10.1163/156856897X00357 9176952

[B9] Calvo-Merino B. Glaser D. E. Grèzes J. Passingham R. E. Haggard P. (2005). Action observation and acquired motor skills: an fMRI study with expert dancers. Cereb. Cortex 15, 1243–1249. 10.1093/cercor/bhi007 15616133

[B10] Casile A. Giese M. A. (2006). Nonvisual motor training influences biological motion perception. Curr. Biol. 16, 69–74. 10.1016/j.cub.2005.10.071 16401424

[B11] Croft R. J. Barry R. J. (2000). Removal of ocular artifact from the EEG: a review. Neurophysiol. Clin. 30, 5–19. 10.1016/S0987-7053(00)00055-1 10740792

[B12] Delorme A. (2006). Statistical methods, in Encyclopedia of Medical Device and Instrumentation, Vol 6, ed Webster J. G. (Wiley), 240–264.

[B13] Downing P. E. Jiang Y. Shuman M. Kanwisher N. (2001). A cortical area selective for visual processing of the human body. Science 293, 2470–2473. 10.1126/science.1063414 11577239

[B14] Fling B. W. Cohen R. G. Mancini M. Carpenter S. D. Fair D. A. Nutt J. G.. (2014). Functional reorganization of the locomotor network in Parkinson patients with freezing of gait. PLoS ONE 9:e100291. 10.1371/journal.pone.0100291 24937008 PMC4061081

[B15] Gallese V. Fadiga L. Fogassi L. Rizzolatti G. (1996). Action recognition in the premotor cortex. Brain 119, 593–609. 10.1093/brain/119.2.593 8800951

[B16] Gramfort A. Papadopoulo T. Olivi E. Clerc M. (2011). Forward field computation with OpenMEEG. Comput. Intell. Neurosci. 2011:923703. 10.1155/2011/923703 21437231 PMC3061324

[B17] Grosbras M.-H. Beaton S. Eickhoff S. B. (2012). Brain regions involved in human movement perception: a quantitative voxel-based meta-analysis. Hum. Brain Mapp. 33, 431–454. 10.1002/hbm.21222 21391275 PMC6869986

[B18] Hirai M. Fukushima H. Hiraki K. (2003). An event-related potentials study of biological motion perception in humans. Neurosci. Lett. 344, 41–44. 10.1016/S0304-3940(03)00413-0 12781917

[B19] Hirai M. Senju A. Fukushima H. Hiraki K. (2005). Active processing of biological motion perception: an ERP study. Brain Res. Cogn. Brain Res. 23, 387–396. 10.1016/j.cogbrainres.2004.11.005 15820645

[B20] Hiris E. (2007). Detection of biological and nonbiological motion. J. Vis. 4, 1–16. 10.1167/7.12.4 17997646

[B21] Iseki K. Hanakawa T. Shinozaki J. Nankaku M. Fukuyama H. (2008). Neural mechanisms involved in mental imagery and observation of gait. Neuroimage 41, 1021–1031. 10.1016/j.neuroimage.2008.03.010 18450480

[B22] Jastorff J. Orban G. A. (2009). Human functional magnetic resonance imaging reveals separation and integration of shape and motion cues in biological motion processing. J. Neurosci. 29, 7315–7329. 10.1523/JNEUROSCI.4870-08.2009 19494153 PMC6666481

[B23] Jeannerod M. (2001). Neural simulation of action: a unifying mechanism for motor cognition. Neuroimage 14, S103–S109. 10.1006/nimg.2001.0832 11373140

[B24] Johansson G. (1973). Visual perception of biological motion and a model for its analysis. Percept. Psychophys. 14, 201–211. 10.3758/BF03212378

[B25] Jokisch D. Daum I. Suchan B. Troje N. F. (2005). Structural encoding and recognition of biological motion: evidence from event-related potentials and source analysis. Behav. Brain Res. 157, 195–204. 10.1016/j.bbr.2004.06.025 15639170

[B26] Kilner J. M. (2011). More than one pathway to action understanding. Trends Cogn. Sci. 15, 352–357. 10.1016/j.tics.2011.06.005 21775191 PMC3389781

[B27] Krakowski A. I. Ross L. A. Snyder A. C. Sehatpour P. Kelly S. P. Foxe J. J. (2011). The neurophysiology of human biological motion processing: a high-density electrical mapping study. Neuroimage 56, 373–383. 10.1016/j.neuroimage.2011.01.058 21276862 PMC6589837

[B28] Lajoie K. Drew T. (2007). Lesions of area 5 of the posterior parietal cortex in the cat produce errors in the accuracy of paw placement during visually guided locomotion. J. Neurophysiol. 97, 2339–2354. 10.1152/jn.01196.2006 17215501

[B29] Lange J. Pavlidou A. Schnitzler A. (2015). Lateralized modulation of beta-band power in sensorimotor areas during action observation. Front. Integr. Neurosci. 9:43. 10.3389/fnint.2015.00043 26161072 PMC4479727

[B30] Makin A. D. J. Poliakoff E. Ackerley R. El-Deredy W. (2012). Covert tracking: a combined ERP and fixational eye movement study. PLoS ONE 7:e38479. 10.1371/journal.pone.0038479 22719893 PMC3374826

[B31] Malouin F. Richards C. L. Jackson P. L. Dumas F. Doyon J. (2003). Brain activations during motor imagery of locomotor-related tasks: a PET study. Hum. Brain Mapp. 19, 47–62. 10.1002/hbm.10103 12731103 PMC6872050

[B32] Maris E. Oostenveld R. (2007). Nonparametric statistical testing of EEG- and MEG-data. J. Neurosci. Methods 164, 177–190. 10.1016/j.jneumeth.2007.03.024 17517438

[B33] Matheson H. E. McMullen P. A. (2010). Neuropsychological dissociations between motion and form perception suggest functional organization in extrastriate cortical regions in the human brain. Brain Cogn. 74, 160–168. 10.1016/j.bandc.2010.07.009 20727650

[B34] Mulder T. (2007). Motor imagery and action observation: cognitive tools for rehabilitation. J. Neural. Transm. 114, 1265–1278. 10.1007/s00702-007-0763-z 17579805 PMC2797860

[B35] Orgs G. Dovern A. Hagura N. Haggard P. Fink G. R. Weiss P. H. (2015). Constructing visual perception of body movement with the motor cortex. Cereb. Cortex 26, 440–449. 10.1093/cercor/bhv262 26534907 PMC4677987

[B36] Pavlidou A. Schnitzler A. Lange J. (2014). Interactions between visual and motor areas during the recognition of plausible actions as revealed by magnetoencephalography. Hum. Brain Mapp. 35, 581–592. 10.1002/hbm.22207 23117670 PMC6869263

[B37] Peelen M. V. Wiggett A. J. Downing P. E. (2006). Patterns of fMRI activity dissociate overlapping functional brain areas that respond to biological motion. Neuron 49, 815–822. 10.1016/j.neuron.2006.02.004 16543130

[B38] Pozzo T. Papaxanthis C. Petit J. L. Schweighofer N. Stucchi N. (2006). Kinematic features of movement tunes perception and action coupling. Behav. Brain Res. 169, 75–82. 10.1016/j.bbr.2005.12.005 16430976

[B39] Rizzolatti G. Fogassi L. Gallese V. (2001). Neurophysiological mechanisms underlying the understanding and imitation of action. Nat. Rev. Neurosci. 2, 661–670. 10.1038/35090060 11533734

[B40] Saunier G. Martins E. F. Dias E. C. de Oliveira J. M. Pozzo T. Vargas C. D. (2013). Electrophysiological correlates of biological motion permanence in humans. Behav. Brain Res. 236, 166–174. 10.1016/j.bbr.2012.08.038 22964139

[B41] Saunier G. Papaxanthis C. Vargas C. D. Pozzo T. (2008). Inference of complex human motion requires internal models of action: behavioral evidence. Exp. Brain Res. 185, 399–409. 10.1007/s00221-007-1162-2 17955225

[B42] Saygin A. P. (2007). Superior temporal and premotor brain areas necessary for biological motion perception. Brain 130, 2452–2461. 10.1093/brain/awm162 17660183

[B43] Tadel F. Baillet S. Mosher J. C. Pantazis D. Leahy R. M. (2011). Brainstorm: a user-friendly application for MEG/EEG analysis. Comput. Intell. Neurosci. 2011:879716. 10.1155/2011/879716 21584256 PMC3090754

[B44] Ulloa E. R. Pineda J. A. (2007). Recognition of point-light biological motion: mu rhythms and mirror neuron activity. Behav. Brain Res. 183, 188–194. 10.1016/j.bbr.2007.06.007 17658625

[B45] Viviani P. Stucchi N. (1992). Biological movements look uniform: evidence of motor-perceptual interactions. J. Exp. Psychol. Hum. Percept. Perform. 18, 603–623. 10.1037/0096-1523.18.3.603 1500865

[B46] White N. C. Fawcett J. M. Newman A. J. (2014). Electrophysiological markers of biological motion and human form recognition. Neuroimage 84, 854–867. 10.1016/j.neuroimage.2013.09.026 24064067

[B47] Winter D. A. (2009). Biomechanics and Motor Control of Human Movement. New York, NY: John Wiley & Sons.

[B48] World Medical Association General Assembly (2008). World Medical Association Declaration of Helsinki, Ethical Principles for Medical Research involving human subject. World Med. J. 54, 120–124.

